# Malaria vaccine efficacy: the difficulty of detecting and diagnosing malaria

**DOI:** 10.1186/1475-2875-6-36

**Published:** 2007-03-26

**Authors:** Wendy Prudhomme O'Meara, B Fenton Hall, F Ellis McKenzie

**Affiliations:** 1 Fogarty International Center, tel 301-496-0426, fax 301-496-8496, NIH, Bethesda, Maryland, USA; 2 National Institute of Allergy and Infectious Diseases, NIH, Rockville, Maryland, USA

## Abstract

New sources of funding have revitalized efforts to control malaria. An effective vaccine would be a tremendous asset in the fight against this devastating disease and increasing financial and scientific resources are being invested to develop one. A few candidates have been tested in Phase I and II clinical trials, and several others are poised to begin trials soon. Some studies have been promising, and others disappointing.

It is difficult to compare the results of these clinical trials; even independent trials of the same vaccine give highly discrepant results. One major obstacle in evaluating malaria vaccines is the difficulty of diagnosing clinical malaria. This analysis evaluates the impact of diagnostic error, particularly that introduced by microscopy, on the outcome of efficacy trials of malaria vaccines and make recommendations for improving future trials.

## Background

Vaccines which protect against infectious diseases have saved many lives in the past 200 years, have allowed the eradication of smallpox and near-eradication of polio, and are widely regarded as extremely cost-effective interventions. Vaccines against many common diseases, such as measles and pertussis, have become part of routine paediatric care and public health programs worldwide.

Malaria is estimated to be the deadliest paediatric disease: hundreds of thousands of children under five succumb to malaria each year. An effective vaccine would be a major advance in public health and is actively being sought. Several candidates have been tested in clinical trials and many more are in various stages of development. Many aspects of vaccine trial design are important in ensuring reliable outcomes, but among the most critical are the choice of primary endpoint and thereby the definition of vaccine efficacy. In practice, clinical endpoints depend on three equally important components of malaria diagnosis – detection, quantification and case definition. The factors which contribute to accuracy in each component, specifically with respect to microscopic diagnosis, and their impact on the calculation of vaccine efficacy for various types of Phase II and III clinical trial endpoints are examined.

### Measuring vaccine efficacy

Malaria vaccine strategies can be categorized by the stage of the parasite life cycle which they target. The target of the vaccine candidate will determine the endpoint that is used to evaluate its efficacy (Table 1 and Additional File 1). Unlike most anti-viral or anti-bacterial vaccines, which induce complete protection from clinical disease in a high percentage of vaccinated individuals, no current malaria vaccine candidates induce complete protection against infection or clinical malaria. This is consistent with natural immunity to malaria which does not completely protect from infection even into adulthood. Malaria vaccine efficacy (VE) will reflect considerable underlying heterogeneity in individual protection and immune response to the vaccine. With most infectious diseases, VE compares the frequency of a specified event in the vaccinated population to that in the unvaccinated population; in each individual the event either does or does not occur[1]. With malaria, VE is likely to reflect a relative frequency of the specified event within an individual as well as within the population; infections and symptoms will continue to occur in those successfully vaccinated, albeit less often and/or with less severity.

**Table 1:** Comparison of endpoints of vaccine trials. The mode of action of the vaccine will determine the type of trial and endpoint that is used to evaluate their efficacy.

Target of vaccine*	Trial	Endpoint	Vaccine efficacy	Type of follow up	References
Pre-erythrocytic	Sporozoite challenge (Phase IIa)	Infection	1−NVNUV MathType@MTEF@5@5@+=feaafiart1ev1aaatCvAUfKttLearuWrP9MDH5MBPbIqV92AaeXatLxBI9gBaebbnrfifHhDYfgasaacH8akY=wiFfYdH8Gipec8Eeeu0xXdbba9frFj0=OqFfea0dXdd9vqai=hGuQ8kuc9pgc9s8qqaq=dirpe0xb9q8qiLsFr0=vr0=vr0dc8meaabaqaciaacaGaaeqabaqabeGadaaakeaacqaIXaqmcqGHsisldaWcaaqaaiabd6eaonaaBaaaleaacqWGwbGvaeqaaaGcbaGaemOta40aaSbaaSqaaiabdwfavjabdAfawbqabaaaaaaa@34E2@	Daily ACD	[30, 31]
		Delayed patency (if vaccinees become infected)	--	Daily ACD	[32–34]
		Number of primary merozoites**	1−P⌢VP⌢UV MathType@MTEF@5@5@+=feaafiart1ev1aaatCvAUfKttLearuWrP9MDH5MBPbIqV92AaeXatLxBI9gBaebbnrfifHhDYfgasaacH8akY=wiFfYdH8Gipec8Eeeu0xXdbba9frFj0=OqFfea0dXdd9vqai=hGuQ8kuc9pgc9s8qqaq=dirpe0xb9q8qiLsFr0=vr0=vr0dc8meaabaqaciaacaGaaeqabaqabeGadaaakeaacqaIXaqmcqGHsisldaWcaaqaaiqbdcfaqzaataWaaSbaaSqaaiabdAfawbqabaaakeaacuWGqbaugaWeamaaBaaaleaacqWGvbqvcqWGwbGvaeqaaaaaaaa@351E@	Daily ACD	[5]
	Field trial in endemic setting (Phase IIb/III)	Time to first infection or first episode	1-HR	ACD (for time to infection) ACD or PCD (for first episodes)	[35, 36]
		Incidence of disease episodes	1−IVIUV MathType@MTEF@5@5@+=feaafiart1ev1aaatCvAUfKttLearuWrP9MDH5MBPbIqV92AaeXatLxBI9gBaebbnrfifHhDYfgasaacH8akY=wiFfYdH8Gipec8Eeeu0xXdbba9frFj0=OqFfea0dXdd9vqai=hGuQ8kuc9pgc9s8qqaq=dirpe0xb9q8qiLsFr0=vr0=vr0dc8meaabaqaciaacaGaaeqabaqabeGadaaakeaacqaIXaqmcqGHsisldaWcaaqaaiabdMeajnaaBaaaleaacqWGwbGvaeqaaaGcbaGaemysaK0aaSbaaSqaaiabdwfavjabdAfawbqabaaaaaaa@34CE@	ACD or PCD	For multiple episodes [36]
Blood stage	Field trial in endemic setting	Incidence of disease episodes (first episode or multiple episodes) (Phase IIb/III)	1−IVIUV MathType@MTEF@5@5@+=feaafiart1ev1aaatCvAUfKttLearuWrP9MDH5MBPbIqV92AaeXatLxBI9gBaebbnrfifHhDYfgasaacH8akY=wiFfYdH8Gipec8Eeeu0xXdbba9frFj0=OqFfea0dXdd9vqai=hGuQ8kuc9pgc9s8qqaq=dirpe0xb9q8qiLsFr0=vr0=vr0dc8meaabaqaciaacaGaaeqabaqabeGadaaakeaacqaIXaqmcqGHsisldaWcaaqaaiabdMeajnaaBaaaleaacqWGwbGvaeqaaaGcbaGaemysaK0aaSbaaSqaaiabdwfavjabdAfawbqabaaaaaaa@34CE@	ACD or PCD	[15, 16, 37]
		Density of infection (Phase IIb)	1−D⌢VD⌢UV MathType@MTEF@5@5@+=feaafiart1ev1aaatCvAUfKttLearuWrP9MDH5MBPbIqV92AaeXatLxBI9gBaebbnrfifHhDYfgasaacH8akY=wiFfYdH8Gipec8Eeeu0xXdbba9frFj0=OqFfea0dXdd9vqai=hGuQ8kuc9pgc9s8qqaq=dirpe0xb9q8qiLsFr0=vr0=vr0dc8meaabaqaciaacaGaaeqabaqabeGadaaakeaacqaIXaqmcqGHsisldaWcaaqaaiqbdseaezaataWaaSbaaSqaaiabdAfawbqabaaakeaacuWGebargaWeamaaBaaaleaacqWGvbqvcqWGwbGvaeqaaaaaaaa@34EE@	ACD	[9]

N = number of volunteers infected, P = mean number of primary merozoites, HR = Hazards ratio, I=incidence, D = mean density, V = vaccinated, UV = unvaccinated, ACD = active case detection, PCD = passive case detection

* Transmission blocking (mosquito stage) vaccines are not considered in this analysis.

** This endpoint requires PCR and is not currently used as a primary endpoint

Vaccine efficacy is calculated as a ratio, which leads to the common misconception that uncertainty (i.e. false positives or false negatives) in the numerator and uncertainty in the denominator will cancel or balance. This is not the case, particularly when sensitivity and specificity may differ in the vaccinated and the control groups. The 95% confidence intervals with which VE is typically reported give the range of values within which one can be 95% certain that the true VE lies, based on the observations, but they reflect only one type of uncertainty. They describe the role of random chance in the observations and depend on the sample size and number of observed disease events. They do not reflect measurement error or other types of uncertainty that may be introduced during the trial. The effect of the uncertainty of the measurement technique on the reliability of VE calculations has not been fully evaluated and must be properly incorporated.

### Detecting malaria

When making measurements with categorical conclusions, such as the presence or absence of parasites, measurement uncertainty can decrease the sensitivity and specificity of the endpoint. Low sensitivity results in a misclassification of true positives as negatives and may arise as a result of measurement error or a case definition that is too stringent. If the ex ante sensitivity is low, it can be counterbalanced by increasing the sample size. However, if the ex post sensitivity (as evaluated after the intervention) is lower in the vaccinated group than in the control group, a systematic bias is introduced and the VE will be overestimated[2].

**Figure 1:**
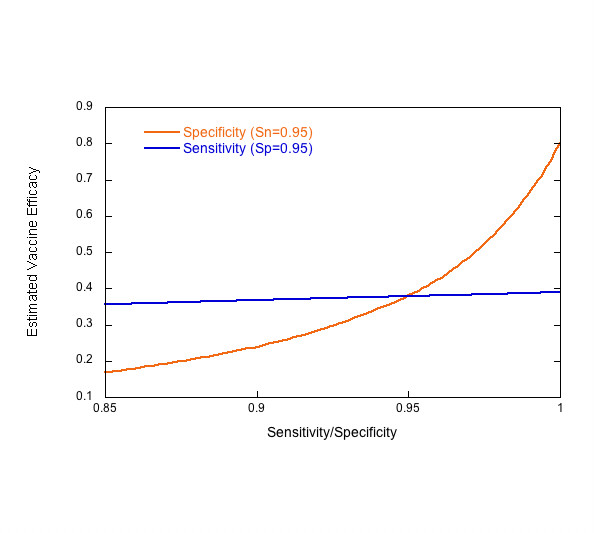
Estimated vaccine efficacy as a function of sensitivity and specificity for a vaccine with a true efficacy of 80% and an attack rate of 5%. Adapted from [3].

False positives may be generated by measurement error or a case definition that is too inclusive. Specificity less than 100% can have a dramatic impact on the ability to accurately measure VE[3] (Figure 1). For example, a decrease of 5% in specificity, from 95% to 90%, can reduce the estimated VE by 50% for an attack rate of 5% in the control group. The impact of specificity on VE becomes important when the number of false positives becomes a significant proportion of the total observed positives and increases as the attack rate declines. In addition, decreased specificity biases the relative risk in the intervention group towards 1.0, which obscures the difference between vaccine candidates[2]. This will become especially relevant when the first malaria vaccine is licensed, after which placebo-controlled trials will likely be impossible and Phase IIb and III trials will be designed to compare between two vaccines.

**Figure 2:**
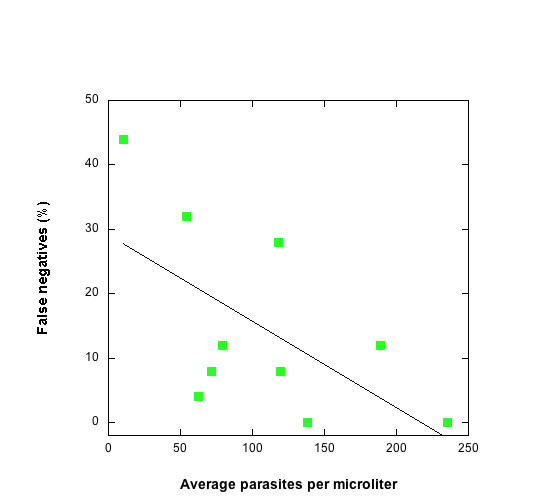
The sensitivity of microscopy as a function of parasite density. The percentage of microscopists (n = 25) reporting a false negative slide versus the mean parasite density reported by the remaining microscopists (adapted from [38]).

Classification of an individual as parasite-negative or -positive by microscopy is not a true binary measurement. A parasite-negative slide is one which has a parasite density below the sensitivity, or limit of detection (sensitivity) of microscopy. The LOD is operator-dependent, but is also correlated with parasite density and has been shown to be highly variable at low densities (Figure 2) [4]. This points to the difficulty of dealing with a source of uncertainty that scales directly with the success of the intervention: if vaccination reduces the parasite density, then the sensitivity will be lower in the vaccinated cohort.

**Figure 3:**
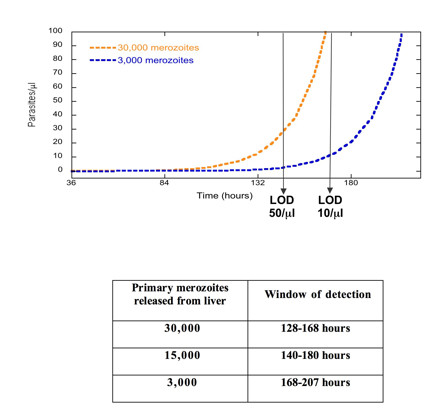
Variable sensitivity can obscure differences between vaccinated and unvaccinated individuals in challenge studies. If blood smears are read by two different microscopists at time = t_1 _and time = t_2_, differences in sensitivity could result in no difference seen between the two study subjects. Microscopist A with an LOD of 50 p/μl would read negative smears at time t_1_. Microscopist B with an LOD of 10 p/μl would read positive smears at time t_2_. An initial number of merozoites are released from the liver at time t = 0 and assumed to grow exponentially with 16 merozoites produced from a single merozoite every 48 hours. The table inset gives the "window of detection" of blood-stage infection when the sensitivity varies between 10 and 100 parasites per microliter for different numbers of primary merozoites released from the liver.

Variable sensitivity at low parasite densities complicates the interpretation of Phase IIa challenge studies for pre-erythrocytic vaccines (Figure 3). Vaccinated and unvaccinated individuals are bitten by a predetermined number of infected mosquitoes. Blood smears are taken at least once per day to detect the first appearance of parasites. A delay in the appearance of parasites in the vaccinated group, relative to the unvaccinated group, is interpreted as a reduction in the number of surviving pre-erythrocytic parasites[5]. Figure 3 plots parasite density as a function of time, assuming simple exponential growth kinetics of the blood-stage infection, for 30,000 and 3,000 primary merozoites released from the liver. The curves are always parallel. Therefore, if the LOD is constant, the delay in detection of parasites in the vaccinated individual will be independent of the LOD. However, if the LOD is variable, then one microscopist may see zero parasites at time (t) in either sample, but a second microscopist, with a lower LOD, may see parasites in both vaccinated and unvaccinated individuals at time (t+Δt), leading to the erroneous conclusion that there was no delay in the appearance of parasites and the vaccine had no effect. For an LOD between 10 and 100/μl, the windows of detection for a 10-fold and 100-fold reduction in primary merozoites overlap (Figure 3), potentially reducing the observed VE.

Low sensitivity can have a similar effect on Phase IIb trials which measure time to first infection in naturally exposed populations; high rates of false negatives and variable sensitivity can obscure a difference between the vaccinated and unvaccinated groups. False negatives have less serious consequences for trial interpretation when the time to first clinical episodes is measured by passive case detection. If no parasites are detected at first presentation and patients are not treated for malaria, then presumably symptoms will persist until parasites are detected and the case is counted.

The specificity of malaria microscopy is also less than perfect, and rates of false positives can reach 24% [6]. The impact of false positives on malaria chemoprophylaxis trials has been evaluated[7]. The analysis is applicable for clinical trials of pre-erythrocytic vaccines where smears are prepared regularly to detect infection; even a 1% decrease in specificity can reduce the observed protective efficacy by 30%.

### Quantifying malaria

Quantifying malaria by microscopy is a difficult task[8], but the importance of this well-known fact is often overlooked. Accurate density determination is critical for at least two endpoints used in clinical trials of malaria vaccines. Blood-stage vaccines can be evaluated by the reduction in density of the blood-stage infection in the vaccinated versus the control group (Phase IIb)[9], and clinical episodes of malaria for any type of vaccine are often defined by a specific density threshold (Phase IIb, III; see next section). Uncertainty in density determination may reduce both the sensitivity and specificity of these endpoints, and the magnitude of the uncertainty is inversely proportional to the density. When density is altered by the intervention (vaccination), the result is a different magnitude of error in the vaccinated versus the control group.

**Figure 4:**
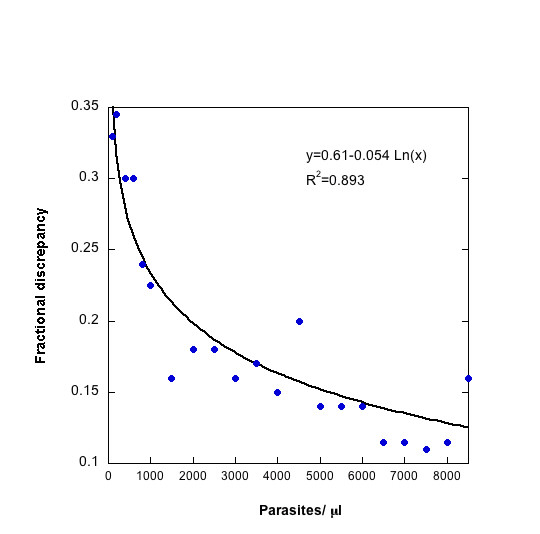
Discrepancy in density between two microscopists reading a single slide as a function of parasite density. Adapted from [8]. Parasite density measurements less than 10,000/μl were assumed to have an error described by the equation: Error = 0.61 – 0.054*Ln(Density). Densities greater than or equal to 10,000/μl were assigned an error of 10%.

In a study conducted in Peru and Thailand, thousands of blood smears were read independently by two microscopists following very detailed protocols[10,11]. The disagreement between the microscopists increased with decreasing density (Figure 4). At very low densities, the median difference was greater than 60%. This relationship between density and discrepancy can be fit with a logarithmic model which describes uncertainty as a continuous function of density. The model can then be used to incorporate the uncertainty associated with each density measurement into the calculation of VE. The difference between the reports from two microscopists reflects the precision in measuring density but does not give any information about accuracy per se and is, therefore, not a measure of distance from true density, but rather of distance between two measurements.

**Figure 5:**
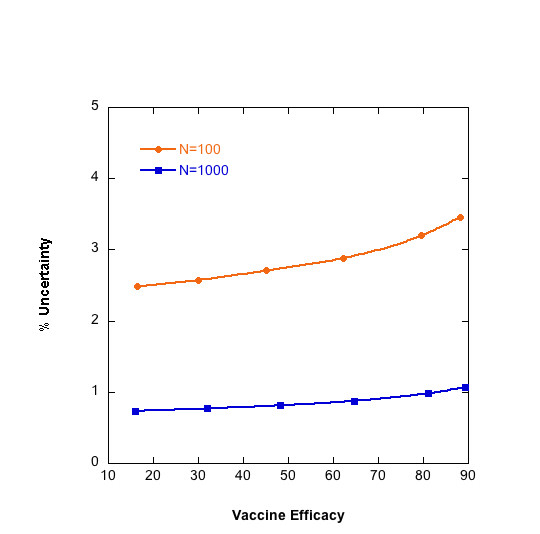
Percent error associated with vaccine efficacy increases as vaccine efficacy increases. Plot shows results for 100 and 1,000 individuals per arm. VE=1−D¯VD¯UV
 MathType@MTEF@5@5@+=feaafiart1ev1aaatCvAUfKttLearuWrP9MDH5MBPbIqV92AaeXatLxBI9gBaebbnrfifHhDYfgasaacH8akY=wiFfYdH8Gipec8Eeeu0xXdbba9frFj0=OqFfea0dXdd9vqai=hGuQ8kuc9pgc9s8qqaq=dirpe0xb9q8qiLsFr0=vr0=vr0dc8meaabaqaciaacaGaaeqabaqabeGadaaakeaacqWGwbGvcqWGfbqrcqGH9aqpcqaIXaqmcqGHsisldaWcaaqaamaanaaabaGaemiraqeaamaaBaaaleaacqWGwbGvaeqaaaGcbaWaa0aaaeaacqWGebaraaWaaSbaaSqaaiabdwfavjabdAfawbqabaaaaaaa@382A@ D¯V
 MathType@MTEF@5@5@+=feaafiart1ev1aaatCvAUfKttLearuWrP9MDH5MBPbIqV92AaeXatLxBI9gBaebbnrfifHhDYfgasaacH8akY=wiFfYdH8Gipec8Eeeu0xXdbba9frFj0=OqFfea0dXdd9vqai=hGuQ8kuc9pgc9s8qqaq=dirpe0xb9q8qiLsFr0=vr0=vr0dc8meaabaqaciaacaGaaeqabaqabeGadaaakeaadaqdaaqaaiabdseaebaadaWgaaWcbaGaemOvayfabeaaaaa@2F2F@ is the mean density in the vaccinated group and D¯UV
 MathType@MTEF@5@5@+=feaafiart1ev1aaatCvAUfKttLearuWrP9MDH5MBPbIqV92AaeXatLxBI9gBaebbnrfifHhDYfgasaacH8akY=wiFfYdH8Gipec8Eeeu0xXdbba9frFj0=OqFfea0dXdd9vqai=hGuQ8kuc9pgc9s8qqaq=dirpe0xb9q8qiLsFr0=vr0=vr0dc8meaabaqaciaacaGaaeqabaqabeGadaaakeaadaqdaaqaaiabdseaebaadaWgaaWcbaGaemyvauLaemOvayfabeaaaaa@3062@ is the mean density in the unvaccinated group. The frequency of densities in a cohort is described by a gamma distribution with a mean density of 3,000 parasites per microliter in the unvaccinated group and 1,500 parasites per microliter in the vaccinated group. Error associated with each density measurement is calculated using the error model in Figure 4 and propagated in the calculation of vaccine efficacy using standard error propagation methods.

When comparing parasite densities in the vaccinated and the control group, uncertainty arising from quantification of the parasite density can be introduced directly into the calculation of VE. Figure 5 shows the results of this simulation. As VE increases, the mean density in the numerator (vaccinated group) decreases and the associated uncertainty increases. Therefore, the percent uncertainty increases as VE increases. However, the magnitude of the uncertainty decreases as the sample size increases. If VE is greater than 80%, an uncertainty of 3% (in the case of 100 individuals in each group) is unlikely to be of serious concern. At intermediate efficacies (40–50%), a 3–4% uncertainty could obscure the difference between successive iterations of a vaccine over the course of its development, leading to unwarranted rejection or pursuit of a vaccine candidate.

Factors other than measurement error can contribute to uncertainty in parasite density measurements. For example, parasite density is reported as parasites per microliter of blood, but because parasites are counted using white blood cells (WBCs) as an index, a conversion factor -generally a uniform approximation of 8,000 WBCs/μl[12]- is used to convert the number of parasites per WBC into parasites per microliter. However, WBC counts may vary with age, infection status and other factors. Counts can be > 13,000 WBC/μl in asymptomatically infected infants and as low as 5,400 WBC/μl in asymptomatically infected adults[13]. WBC counts in malaria-infected, febrile adults are significantly lower than in uninfected, febrile adults[14]. Thus, adopting a single, uniform approximation as a conversion factor can obscure the true parasite density and potentially lead to even larger errors than the pure counting errors described above- e.g. over-estimating parasite densities by 30% relative to true WBC counts[14]. Using complete blood counts to determine the per-patient WBC count or counting by volume, as has been done in some studies (i.e. [9,15-17]), can reduce the problem of patient-to-patient variation in WBC counts.

Temporal fluctuations in parasite density in peripheral blood are common and may have greater consequences for parasite density measurement than uncertainty associated with microscopic quantification. Peaks and troughs of density may arise from sequestration, or immune response and the rise of new antigenic variants. Thus it is seldom clear how to interpret a single measurement of parasite density in an individual. For example, infections quantified 24–48 hours apart in each of 11 patients prior to drug treatment showed an average increase of 5,500 parasites per microliter, with a range of -17,000 to 46,000[18]. A longitudinal study of adults in highly endemic areas reported frequent fluctuations of 100-fold in parasite density within as few as 6 hours[19]. Similar results were seen in children monitored daily[20]. More studies are necessary to determine whether a single density determination is an appropriate measurement endpoint for clinical trials.

It is worth noting that body temperature can also fluctuate on the timescale of hours. Choosing a temperature threshold to define a febrile malaria episode raises similar issues to choosing a single density threshold.

### Defining malaria

In malaria-endemic areas with moderate to high transmission, the presence of parasites alone or parasites with fever are not adequate indicators of a clinical malaria episode. It is not possible to definitively diagnose clinical malaria; therefore, criteria must be chosen for which a patient will be considered to be experiencing a clinical malaria episode. The uncertainty in definition will lead to misclassification of cases, both false positives and false negatives, resulting in decreased sensitivity and specificity.

The fraction of parasitaemic, symptomatic individuals whose malaise is caused by parasites is referred to as the attributable fraction. The attributable fraction can be calculated by comparing the proportion of febrile individuals over a range of parasite densities. Most commonly, a logistic regression model is fit to the data[21] to describe the probability that a fever can be attributed to malaria at any density. The overall attributable fraction is calculated by averaging this probability over all cases. The logistic regression model can be used to calculate the sensitivity and specificity of a particular cut-off density by estimating the number of true cases that will not be counted because parasite density falls below the threshold (false negatives) and the number of non-malaria febrile cases that will be counted as positive because the density exceeds the threshold (false positives). Cases of malaria are then defined by a particular cut-off density which gives the required sensitivity and specificity. As parasite density decreases, the probability that symptoms are due to the observed parasitemia decreases. Therefore, the specificity of the case definition decreases with decreasing threshold density, and the observed vaccine efficacy should also decrease.

The logistic regression model for determining attributable fraction has been used to determine the sensitivity and specificity of case definitions in numerous epidemiological settings. The cut-off density which gives a case definition with sufficiently high sensitivity and specificity depends on many interrelated variables. The appropriate case definition changes as a function of transmission intensity[22], and, therefore, season [23]. Age, which together with transmission intensity determines previous exposure and immunity, has been correlated to changes in attributable fraction [13, 22, 23, 24], and sensitivity and specificity of a particular case definition. Vaccination is designed to alter the immune status, therefore, the case definition will necessarily have different sensitivities and specificities in the vaccinated compared to the unvaccinated group. All of these factors complicate comparisons between trials that use density cut-off case definitions.

**Figure 6:**
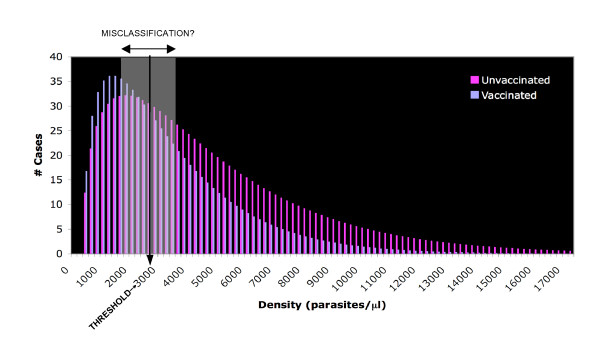
Hypothetical density distribution of clinical episodes of malaria. The distribution of parasite densities among febrile cases is approximated by a gamma distribution with a mean of 3,000/μl in the unvaccinated group and vaccination reduces both the incidence of fever (25% reduction) and the mean parasite density (30% reduction) among the cohort. A cut-off value of 3,000 parasites per microliter is shown and the shaded area represents the cases which risk being misclassified due to the discrepancy between microscopists.

**Figure 7:**
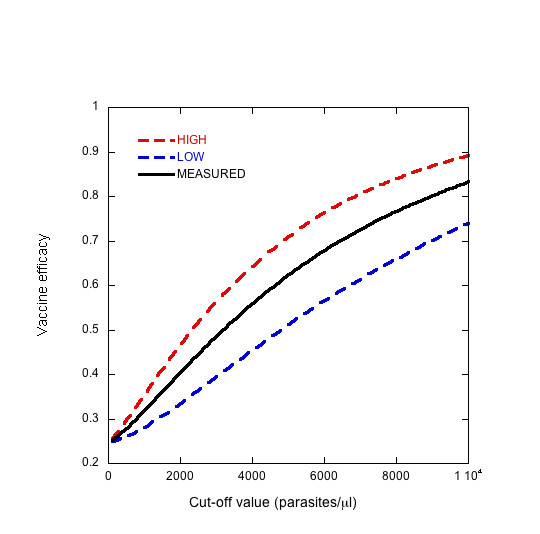
The observed vaccine efficacy (solid line) and the upper (red dashed line) and lower (blue dashed line) limit of possible outcomes as a function of threshold density based on the uncertainty associated with measurement of parasite density. Frequency of cases by density is assumed to follow the distribution in Figure 6. The upper and lower bounds are calculated using the error model in Figure 4.

Once a threshold density has been chosen to define a clinical episode of malaria, the effect of measurement uncertainty inherent in detecting and quantifying the infection can be evaluated. Using the density error model, the fraction of cases which are at risk of being mis-categorized based on the cut-off value can be calculated (Figure 6). The discrepancy between microscopists is assumed to be symmetric around the true density, which is a conservative assumption. These cases could be either mistakenly included or excluded, so the highest and lowest vaccine efficacy that can be expected is calculated for a range of threshold choices. Figure 7 shows the VE that would be measured with no errors as well as the maximum and minimum VE that may be observed as determined by the measurement uncertainty around the threshold density. The upper and lower bounds are theoretical limits, for which all the cases that lie within a certain distance of the threshold are over-counted in one group and under-counted in the other. It is a highly improbable scenario when error is randomly distributed, but biased error, such as an individual microscopist consistently reading higher or lower than others, could shift the density curve in a systematic way. As expected, the VE increases with increasing threshold density and, therefore, increasing specificity of the case definition. In this particular example, for a case definition of fever and > 3,000 p/ml, the VE without measurement error would be 50%, but, based only on the uncertainty associated with accurately measuring parasite density, a VE between 40% and 60% may be observed. These results highlight the risk that a promising vaccine candidate will be unduly abandoned.

### How to reduce uncertainty?

Understanding the sources and impact of uncertainty can lead to more robust endpoint definitions in malaria vaccine trials. The sensitivity and specificity of microscopy are highly reader-dependent. However, particularly in the case of sensitivity, there are intrinsic limitations to microscopy that cannot be completely resolved by improving reader performance. The shape of the density-error relationship amongst the study microscopists should be determined before the study begins. "Over-reading" paradigms, for example having two microscopists read every slide and a third microscopist re-read slides with highly discrepant results, increase the sensitivity and specificity of microscopy and allow on-going, 'real-time' evaluation of the density-error relationship.

Density-ratio endpoints seem to be less sensitive to uncertainty than threshold-dependent endpoints, particularly when the majority of cases fall close to the threshold value. To reduce the error of a threshold-dependent endpoint, a cut-off value based on the parasite:leukocyte ratio rather than an absolute density may be used. Evidence indicates that the parasite:leukocyte ratio is bimodal [13, 25], giving a natural choice for a cut-off value. Deviations from this cutoff value due to experimental error would result in fewer misclassified cases. This observation is consistent with data from other studies which showed that WBC counts were lower in symptomatic, infected individuals compared to those with similar symptoms but no parasites [14, 26].

Another alternative is to use attributable fraction estimates, arrived at by logistic regression[22, 27, 28] or other methods [29], to evaluate efficacy. A reduction in the febrile episodes attributable to malaria across all densities would be a more accurate indicator of vaccine efficacy than defining a clinical episode by a cut-off threshold and would partially eliminate the problem of differing sensitivities and specificities in each arm of the trial. Because vaccination is intended to alter immune status, the relationship between density and probability of fever must be determined by a cross-sectional survey or continuous active detection in the vaccinated and unvaccinated cohorts separately. Each febrile episode recorded during the trial is assigned a probability of being a malaria episode, as a function of its density using the logistic regression model [28]. The number of febrile cases attributable to malaria in the vaccinated and unvaccinated cohorts is calculated by summing the relative risk for febrile episodes in each cohort and multiplying by the total number of febrile cases. This definition of vaccine efficacy reflects both the reduction in parasite density and the reduction in the total number of febrile episodes. It allows for changes in the relationship between parasitemia and clinical illness. Although density measurement is required, this technique is less sensitive to error in individual density measurements because the logistic regression model represents smoothing of aggregated data and as such is less sensitive to random measurement error (Table 2). Furthermore, assigning risk on a continuous scale for a particular parasite density avoids the problem of assigning a binary outcome (clinical episode of malaria or not) based on a continuous, population-averaged model. Vaccine efficacy based on attributable fraction estimates may facilitate comparison between trials in different epidemiological settings and study populations. A comparison of the relationship between probability of fever and density in the vaccinated and the unvaccinated group could give additional insight into the mode of action of the vaccine.

**Table 2:** Errors in density measurement have little impact on vaccine efficacy calculated by attributable fraction.

Mode of action of vaccine	True VE	Lower limit	Upper limit
30% reduction in density 25% reduction in febrile episodes	39.8	39.3	40.3
50% reduction in density 50% reduction in febrile episodes	89.5	89.5	89.5
70% reduction in density 25% reduction in febrile episodes	89.4	88.9	89.8

VE=1−[AFV×NVAFUV×NUV]
 MathType@MTEF@5@5@+=feaafiart1ev1aaatCvAUfKttLearuWrP9MDH5MBPbIqV92AaeXatLxBI9gBaebbnrfifHhDYfgasaacH8akY=wiFfYdH8Gipec8Eeeu0xXdbba9frFj0=OqFfea0dXdd9vqai=hGuQ8kuc9pgc9s8qqaq=dirpe0xb9q8qiLsFr0=vr0=vr0dc8meaabaqaciaacaGaaeqabaqabeGadaaakeaacqWGwbGvcqWGfbqrcqGH9aqpcqaIXaqmcqGHsisldaWadaqaamaalaaabaGaemyqaeKaemOray0aaSbaaSqaaiabdAfawbqabaGccqGHxdaTcqWGobGtdaWgaaWcbaGaemOvayfabeaaaOqaaiabdgeabjabdAeagnaaBaaaleaacqWGvbqvcqWGwbGvaeqaaOGaey41aqRaemOta40aaSbaaSqaaiabdwfavjabdAfawbqabaaaaaGccaGLBbGaayzxaaaaaa@46A3@, where AF is the attributable fraction, N is the total number of febrile episodes and V and UV designate vaccinated and unvaccinated groups, respectively. The vaccine efficacy in the absence of measurement error and the upper and lower bounds that may be observed due to measurement error are reported for different levels of reduction in parasite density and number of febrile episodes. The data from Smith *et al *[21] are used to fit the logistic regression model of the probability of fever as a function of parasite density. Density data is simulated by randomly choosing cases from specific density categories using a very large data set of febrile patients in order to match the proportions of the above data set.

## Conclusion

To obtain accurate results, maximize the ability to compare and distinguish between vaccine candidates, and avoid scuttling promising candidates, it is essential to validate the choice of endpoint and the sensitivity and specificity of the endpoint. Measurement error has a significant impact on the quality and reliability of the outcome and should be considered when developing clinical trial protocols. The type of endpoint chosen determines the extent to which measurement error may affect the calculation of VE, ranging from completely obscuring the true efficacy to differences of a few percentage points. Calculating VE using attributable fraction may reduce the error introduced by counting parasites and improve the ability to compare between vaccine trials.

## Competing interests

The author(s) declare that they have no competing interests.

## Authors' contributions

WPO performed analysis, contributed to intellectual content and drafting of manuscript. BFH contributed to intellectual content and drafting of manuscript. FEM contributed to study design, intellectual content and drafting of manuscript.

## Supplementary Material

Additional file 1 Discussion of malaria detection and diagnostic techniques and alternative endpoints such as severe disease and mortality. Click here for file
