# Supplementary material for: Malaria vaccine efficacy: the difficulty of detecting and diagnosing malaria
Source: Malar J. 2007 Mar 26;6:36. doi: 10.1186/1475-2875-6-36 (PMC1852320; doi:10.1186/1475-2875-6-36)
Supplement: Additional file 1 — Discussion of malaria detection and diagnostic techniques and alternative endpoints such as severe disease and mortality. [file 1475-2875-6-36-S1.doc]

**Supplemental material for Prudhomme O’Meara *et al* “Malaria vaccine efficacy: the difficulty of detecting and diagnosing malaria”**

**I. Types of malaria vaccines**

Malaria vaccine strategies can be categorized by their intended primary mode of protection and the stage of the parasite lifecycle which they target. The mode of action of the vaccine candidate will determine the type of trial that is used to evaluate their efficacy (Also see Table 1 of the main text).

*PRE-ERYTHROCYTIC*

Pre-erythrocytic vaccines are designed to prevent the establishment of the liver-stage of the malaria infection (and thus the subsequent release of primary merozoites into the blood) by targeting either the blood-borne sporozoites or the infected hepatocytes. These types of vaccines can be evaluated in challenge studies during which volunteers with no history of previous malaria infection are immunized and then challenged with sporozoites, along with their unimmunized counterparts (phase IIa trials). It is currently not possible to directly measure the number of sporozoites which establish in the liver, nor is it possible to quantify the number of primary merozoites released; therefore, the appearance of the blood-stage infection must serve as a proxy. In the case of a perfect vaccine, the blood-stage infection should be completely absent, somewhat simplifying the trial. This is seldom if ever the case, however. Instead, a delay in the appearance of a blood-stage infection in vaccinated individuals is interpreted to mean that fewer sporozoites were able to invade and differentiate in the liver. Experimental evidence supports this as the mode of action of at least two liver stage vaccines, FP9 and RTS,S, rather than reduced growth rate in the blood or increased duration of the liver stage [1]. Vaccine efficacy is defined as one minus the ratio of the individuals who remain parasite-free for a specified period of time (usually 21-60 days) in the vaccinated (N_v_) over the unvaccinated group (N_uv_) [2,3]. In the event that some of the vaccinees are not completely protected, the average time to detection of the infection (delayed patency) in the vaccinated group is compared to that of the unvaccinated group [4-6]. This may become the primary endpoint if most (or all) of the vaccinees develop blood-stage infections.

If challenge studies are successful, it is then necessary to evaluate the safety and efficacy of the vaccine under conditions of natural inoculation in individuals with some existing immunity or exposure to the parasite (phase IIb trial) - in other words, in situations and populations where the vaccine may be deployed if it becomes licensed. Because the exact time of inoculation, or infectious mosquito bite, is not known, days to blood-stage patency cannot be used as a measure of vaccine efficacy. Instead, volunteers may be treated to ensure that any existing malaria parasites are completely cleared from both the blood and the liver. They are vaccinated and then followed by both active and passive case detection along with a control cohort [7,8]. The time to the first parasitemia in each individual is recorded and the groups are compared by survival analysis, typically by calculating the hazards ratio using Cox regression. Vaccine efficacy is defined as one minus the hazards ratio. The time to the first clinical episode can be evaluated similarly by replacing active surveillance with passive detection. A significant delay in the time to first clinical episode and vaccine efficacy are calculated as above [9].

*BLOOD-STAGE*

Blood-stage vaccines require a different approach than pre-erythrocytic vaccines. Challenge studies are difficult, particularly because the volunteers must be monitored closely and treated at the first microscopic detection of parasites, minimizing the contact between the immune system and the blood-stage infection. In a blood-stage challenge model proposed by Cheng *et al* [10], individuals were infected by injecting cultured parasites, parasite density was quantified by PCR over several days, and the growth rates were calculated based on the time-series data. The number of parasites was too low to be detectable by microscopy and all volunteers remained asymptomatic until the time curative drug was administered. This model was used to evaluate a 3-part recombinant blood-stage vaccine [11], but no differences in the growth rate of the parasites in the vaccinated group compared to the control could be measured. This approach has not been validated in other trials.

A perfect, anti-parasitic vaccine would completely prevent blood-stage infection, making the presence or absence of parasites an acceptable measure of vaccine performance. However, in the absence of a perfect vaccine, the ability of the vaccine to reduce parasite load [12]or reduce morbidity from infection (i.e. clinical episodes) can be measured. Clinical episodes of malaria may be defined as any signs or symptoms of malaria accompanied by any parasitemia, or fever accompanied by parasitemia, or fever accompanied by parasitemia in excess of a particular threshold value [13-16].

*SEXUAL STAGE*

Malaria vaccines which target the sexual stage of the parasite, also called transmission-blocking vaccines, are being developed to interrupt the parasite life cycle. In this case, there would be no immediate health benefit to the vaccinated individual. Reduced transmission may lead to reduced morbidity and mortality in the community, but the extent of this effect will depend heavily on what fraction of the total population is immunized. The endpoints which must be used to measure the efficacy of a transmission-blocking vaccine are substantially different than those for the vaccines described above and are not discussed in this analysis.

**II. Alternative methods to microscopy**

Parasite DNA or RNA can be detected by polymerase-chain reaction (PCR). PCR is very sensitive when compared to microscopy, but still prone to false positives (2% false positives[17]). However, sensitivity and specificity can be calibrated based on internal positive and negative controls and are less dependent on operator performance than microscopy. Interestingly, one study demonstrated a much lower limit of detection for real-time PCR than for microscopy, but highly variable sensitivity and density determination below a certain threshold [18], similar to microscopy. To circumvent this problem, the investigators chose a cut-off value of 20 parasites per milliliter and classified PCR-positive samples below this threshold as negative. In addition, they calculated delay to parasitemia (or vaccine efficacy) as the days until PCR results reached greater than 1000 p/ml, thereby eliminating the highly variable results observed at very low densities. This is not possible for challenge studies using microscopy; participants must be treated upon detection of the first parasite.

Another advantage of quantitative real-time PCR in challenge studies is that the number of merozoites released can be calculated from the time series of PCR results [1,19], a much more direct measurement of efficacy than appearance of the blood-stage infection. This is also not possible for microscopy, for the reason stated above.

PCR may not be appropriate for trials in which detection of parasites is used to define a clinical episode; submicroscopic infections which are detectable only by PCR are not likely to be clinically relevant. However, PCR can provide valuable secondary endpoint information such as multiplicity of infection, duration of infection, and the presence of cryptic mixed infections.

Rapid immunochromatographic techniques, or dipsticks, are less sensitive and specific than PCR or microscopy and have variable sensitivity at low densities [20]. They give much more rapid results than microscopy, but they cannot distinguish between all species of *Plasmodium*, nor can they be used to determine parasite density. As a result, they are likely to have limited value in vaccine trials.

In vitro culture methods, which use blood samples from inoculated volunteers and culture them to amplify an infection, have been used in some challenge studies [21], particularly when symptoms indicate malaria but parasites cannot be detected by microscopy. However, the success rate of initiating cultures from human infections is not 100%, and the methods are time-consuming and ultimately rely on microscopy to detect the parasites after culture. The sensitivity of such a technique will depend both on the sensitivity of the microscopic detection and the success rate of initiating cultures in vitro.

**Alternative endpoints to uncomplicated clinical malaria**

Other possible endpoints in addition to episodes of uncomplicated clinical malaria are severe malaria, malaria-specific mortality, and overall mortality. Severe malaria may be a more specific endpoint than fever with parasites, but many symptoms of severe malaria are still ambiguous, and, because it is a relatively rare diagnosis (though not rare enough!), large sample sizes are necessary [22]. Malaria-specific mortality is often difficult to determine when relying on verbal autopsies [23,24] and may not be specific enough or reliable enough for vaccine trials. However, overall mortality is unambiguous and can account for indirect effects of vaccination, such as reducing mortality due to complications arising from concurrent malaria [25]. Measuring overall mortality will require even larger sample sizes than severe malaria and may not be practical, despite the perfect sensitivity and specificity.

1. Bejon P, Andrews L, Andersen RF, Dunachie S, Webster D, et al. (2005) Calculation of liver-to-blood inocula, parasite growth rates, and preerythrocytic vaccine efficacy, from serial quantitative polymerase chain reaction studies of volunteers challenged with malaria sporozoites. Journal of Infectious Diseases 191: 619-626.

2. Stoute JA, Slaoui M, Heppner DG, Momin P, Kester KE, et al. (1997) A preliminary evaluation of a recombinant circumsporozoite protein vaccine against Plasmodium falciparum malaria. New England Journal of Medicine 336: 86-91.

3. Kester KE, McKinney DA, Tornieporth N, Ockenhouse CF, Heppner DG, et al. (2001) Efficacy of recombinant circumsporozoite protein vaccine regimens against experimental Plasmodium falciparum malaria. Journal of Infectious Diseases 183: 640-647.

4. Hoffman SL, Edelman R, Bryan JP, Schneider I, Davis J, et al. (1994) Safety, Immunogenicity, and Efficacy of a Malaria Sporozoite Vaccine Administered with Monophosphoryl Lipid a, Cell-Wall Skeleton of Mycobacteria, and Squalane as Adjuvant. American Journal of Tropical Medicine and Hygiene 51: 603-612.

5. Ockenhouse CF, Sun PF, Lanar DE, Wellde BT, Hall BT, et al. (1998) Phase I/IIa safety, immunogenicity, and efficacy trial of NYVAC-Pf7, a pox-vectored, multiantigen, multistage vaccine candidate for Plasmodium falciparum malaria. Journal of Infectious Diseases 177: 1664-1673.

6. Webster DP, Dunachie S, Vuola JM, Berthoud T, Keating S, et al. (2005) Enhanced T cell-mediated protection against malaria in human challenges by using the recombinant poxviruses FP9 and modified vaccinia virus Ankara. Proceedings of the National Academy of Sciences of the United States of America 102: 4836-4841.

7. Brown AE, Singharaj P, Webster HK, Pipithkul J, Gordon DM, et al. (1994) Safety, Immunogenicity and Limited Efficacy Study of a Recombinant Plasmodium-Falciparum Circumsporozoite Vaccine in Thai Soldiers. Vaccine 12: 102-108.

8. Bojang KA, Milligan PJM, Pinder M, Vigneron L, Alloueche A, et al. (2001) Efficacy of RTS,S/ASO2 malaria vaccine against Plasmodium falciparum infection in semi-immune adult men in The Gambia: a randomised trial. Lancet 358: 1927-1934.

9. Alonso PL, Sacarlal J, Aponte JJ, Leach A, Macete E, et al. (2004) Efficacy of the RTS,S/AS02A vaccine against Plasmodium falciparum infection and disease in young African children: randomised controlled trial. Lancet 364: 1411-1420.

10. Cheng Q, Lawrence G, Reed C, Stowers A, RanfordCartwright L, et al. (1997) Measurement of Plasmodium falciparum growth rates in vivo: A test of malaria vaccines. American Journal of Tropical Medicine and Hygiene 57: 495-500.

11. Lawrence G, Cheng Q, Reed C, Taylor D, Stowers A, et al. (2000) Effect of vaccination with 3 recombinant asexual-stage malaria antigens on initial growth rates of Plasmodium falciparum in non-immune volunteers. Vaccine 18: 1925-1931.

12. Genton B, Betuela I, Felger I, Al-Yaman F, Anders RF, et al. (2002) A recombinant blood-stage malaria vaccine reduces Plasmodium falciparum density and exerts selective pressure on parasite populations in a phase 1-2b trial in Papua New Guinea. Journal of Infectious Diseases 185: 820-827.

13. D'Alessandro U, Leach A, Drakeley CJ, Bennett S, Olaleye BO, et al. (1995) Efficacy Trial of Malaria Vaccine Spf66 in Gambian Infants. Lancet 346: 462-467.

14. Acosta CJ, Galindo CM, Schellenberg D, Aponte JJ, Kahigwa E, et al. (1999) Evaluation of the SPf66 vaccine for malaria control when delivered through the EPI scheme in Tanzania. Tropical Medicine & International Health 4: 368-376.

15. Nosten F, Luxemburger C, Kyle DE, Ballou WR, Wittes J, et al. (1996) Randomised double-blind placebo-controlled trial of SPf66 malaria vaccine in children in northwestern Thailand. Lancet 348: 701-707.

16. Alonso PL, Smith T, Schellenberg J, Masanja H, Mwankusye S, et al. (1994) Randomized Trial of Efficacy of Spf66 Vaccine against Plasmodium-Falciparum Malaria in Children in Southern Tanzania. Lancet 344: 1175-1181.

17. Barker RH, Banchongaksorn T, Courval JM, Suwonkerd W, Rimwungtragoon K, et al. (1994) Plasmodium-Falciparum and Plasmodium-Vivax - Factors Affecting Sensitivity and Specificity of Pcr-Based Diagnosis of Malaria. Experimental Parasitology 79: 41-49.

18. Andrews L, Andersen RF, Webster D, Dunachie S, Walther RM, et al. (2005) Quantitative real-time polymerase chain reaction for malaria diagnosis and its use in malaria vaccine clinical trials. American Journal of Tropical Medicine and Hygiene 73: 191-198.

19. Hermsen CC, De Vlas SJ, Van Gemert GJA, Telgt DSC, Verhage DF, et al. (2004) Testing vaccines in human experimental malaria: Statistical analysis of parasitemia measured by a quantitative real-time polymerase chain reaction. American Journal of Tropical Medicine and Hygiene 71: 196-201.

20. Craig MH, Bredenkamp BL, Williams CHV, Rossouw EJ, Kelly VJ, et al. (2002) Field and laboratory comparative evaluation of ten rapid malaria diagnostic tests. Transactions of the Royal Society of Tropical Medicine and Hygiene 96: 258-265.

21. Church LWP, Le TP, Bryan JP, Gordon DM, Edelman R, et al. (1997) Clinical manifestations of Plasmodium falciparum malaria experimentally induced by mosquito challenge. Journal of Infectious Diseases 175: 915-920.

22. Lyke KE, Dicko A, Kone A, Coulibaly D, Guindo A, et al. (2004) Incidence of severe Plasmodium falciparum malaria as a primary endpoint for vaccine efficacy trials in Bandiagara, Mali. Vaccine 22: 3169-3174.

23. Snow RW, Armstrong JRM, Forster D, Winstanley MT, Marsh VM, et al. (1992) Childhood Deaths in Africa - Uses and Limitations of Verbal Autopsies. Lancet 340: 351-355.

24. Todd JE, Defrancisco A, Odempsey TJD, Greenwood BM (1994) The Limitations of Verbal Autopsy in a Malaria-Endemic Region. Annals of Tropical Paediatrics 14: 31-36.

25. Jaffar S, Leach A, Smith PG, Cutts F, Greenwood B (2003) Effects of misclassification of causes of death on the power of a trial to assess the efficacy of a pneumococcal conjugate vaccine in The Gambia. International Journal of Epidemiology 32: 430-436.
